# Ablation of CNTN2+ Pyramidal Neurons During Development Results in Defects in Neocortical Size and Axonal Tract Formation

**DOI:** 10.3389/fncel.2019.00454

**Published:** 2019-11-01

**Authors:** Maria Eleni Kastriti, Aikaterini Stratigi, Dimitris Mariatos, Marina Theodosiou, Maria Savvaki, Michaela Kavkova, Kostas Theodorakis, Marina Vidaki, Tomas Zikmund, Jozef Kaiser, Igor Adameyko, Domna Karagogeos

**Affiliations:** ^1^Department of Basic Science, Faculty of Medicine, University of Crete, Heraklion, Greece; ^2^Foundation for Research and Technology, Institute of Molecular Biology and Biotechnology, Heraklion, Greece; ^3^Department of Physiology and Pharmacology, Karolinska Institutet, Stockholm, Sweden; ^4^Center for Brain Research, Medical University Vienna, Vienna, Austria; ^5^Laboratory of Neurophysiology, Université Libre de Bruxelles, UNI, Brussels, Belgium; ^6^Department of Molecular Medicine, Max Planck Institute of Biochemistry, Martinsried, Germany; ^7^CEITEC-Central European Institute of Technology, Brno University of Technology, Brno, Czechia; ^8^The Koch Institute for Integrative Cancer Research, Massachusetts Institute of Technology, Cambridge, MA, United States; ^9^Department of Biology, Massachusetts Institute of Technology, Cambridge, MA, United States

**Keywords:** CNTN2/TAG-1, corticofugal system, corticothalamic axons, anterior commissure, corpus callosum, central nervous system

## Abstract

Corticothalamic axons express *Contactin-2* (CNTN2/TAG-1), a neuronal recognition molecule of the immunoglobulin superfamily involved in neurogenesis, neurite outgrowth, and fasciculation. TAG-1, which is expressed transiently by cortical pyramidal neurons during embryonic development, has been shown to be fundamental for axonal recognition, cellular migration, and neuronal proliferation in the developing cortex. Although *Tag-1*^−/−^ mice do not exhibit any obvious defects in the corticofugal system, the role of TAG-1+ neurons during the development of the cortex remains elusive. We have generated a mouse model expressing EGFP under the *Tag-1* promoter and encompassing the coding sequence of Diptheria Toxin subunit A (DTA) under quiescence with no effect on the expression of endogenous *Tag-1*. We show that while the line recapitulates the expression pattern of the molecule, it highlights an extended expression in the forebrain, including multiple axonal tracts and neuronal populations, both spatially and temporally. Crossing these mice to the *Emx1-Cre* strain, we ablated the vast majority of TAG-1+ cortical neurons. Among the observed defects were a significantly smaller cortex, a reduction of corticothalamic axons as well as callosal and commissural defects. Such defects are common in neurodevelopmental disorders, thus this mouse could serve as a useful model to study physiological and pathophysiological cortical development.

## Introduction

The multilayered neocortex evolved in mammals and is responsible for a variety of higher functions, seen for the first time in this phylum. Among these higher cognitive functions is the integration of stimuli from different sensory modalities such as vision and olfaction, the control of motor commands, consciousness, decision-making, and particularly in humans, speech.

For these functions to take place, formation of axonal tracts that interconnect the neocortex with other brain areas or other systems of the central nervous system (CNS) and the organs responsible for the perception of stimuli is of tremendous importance. These axonal tracts are formed by neurons born through sequential steps at the ventricular and subventricular zones of the embryonic brain. This tightly regulated process results in the establishment of the six-layered cortical organization at birth, even though neuronal maturation will be completed in later postnatal stages. Several human syndromes have been attributed to or associated with phenotypic and functional abnormalities of cortical pyramidal neurons, such as in the case of people affected by the syndromes of the autistic spectrum or the Miller Dieker syndrome (also known as type I lissencephaly) (Takashima et al., 1981; Sheen et al., 2006a,b).

CNTN2/TAG-1 is a GPI-linked protein-member of the immunoglobulin superfamily and an axonal adhesion molecule, exhibiting a specific and dynamic expression in several CNS and peripheral nervous system (PNS) subpopulations during development (Dodd et al., 1988; Wolfer et al., 1994). In general, its level of expression is strong on axons and it is transient (hence its original name, Transient Axonal Glycoprotein) (Dodd et al., 1988). In the cortex, it is strongly expressed by corticofugal axons during development and has been extensively used as a specific marker of corticothalamic axons (CTAs). Pioneer neurons of the preplate giving rise to CTAs are TAG-1+ and marginal zone (MZ) cells are also TAG-1+ (Wolfer et al., 1994; Denaxa et al., 2001). *Tag-1*^−/−^ mice do not exhibit any obvious defects in the corticofugal system (Denaxa et al., 2005). However, the role of the TAG-1+ neurons during the development of the cortex has not been directly addressed adequately. One of the few studies considering the role of TAG-1 in neuronal development in the neocortex showed that the interaction of the protein with APP at the embryonic ventricular zone of the forebrain has a negative effect on neurogenesis (Ma et al., 2008). Additionally, TAG-1+ axons may form part of the pathway upon which interneurons migrate tangentially on their way from their place of origin (subpallium) to their final cortical locations (Denaxa et al., 2001).

Here, we report on a novel EGFP mouse transgenic strain (*Tag1*^*loxP*−*EGFP*−*loxP*−*DTA*^) recapitulating the expression of *Tag-1*, which in combination with the appropriate *Cre* or *CreERT2* strains can be used to specifically ablate TAG-1+ neurons and consequently, their axons in various CNS and PNS regions. By using the *Emx1-Cre* strain we were able to ablate the vast majority of TAG-1+ cortical neurons from an early time point and therefore observe changes in cortical development and organization. Thus, this mouse can serve as a useful model to study the development of the cortex and potentially contribute to the understanding of the mechanisms resulting in neuronal cortical abnormalities.

## Materials and Methods

### Mouse Strains and Nomenclature

**Figure 1:**
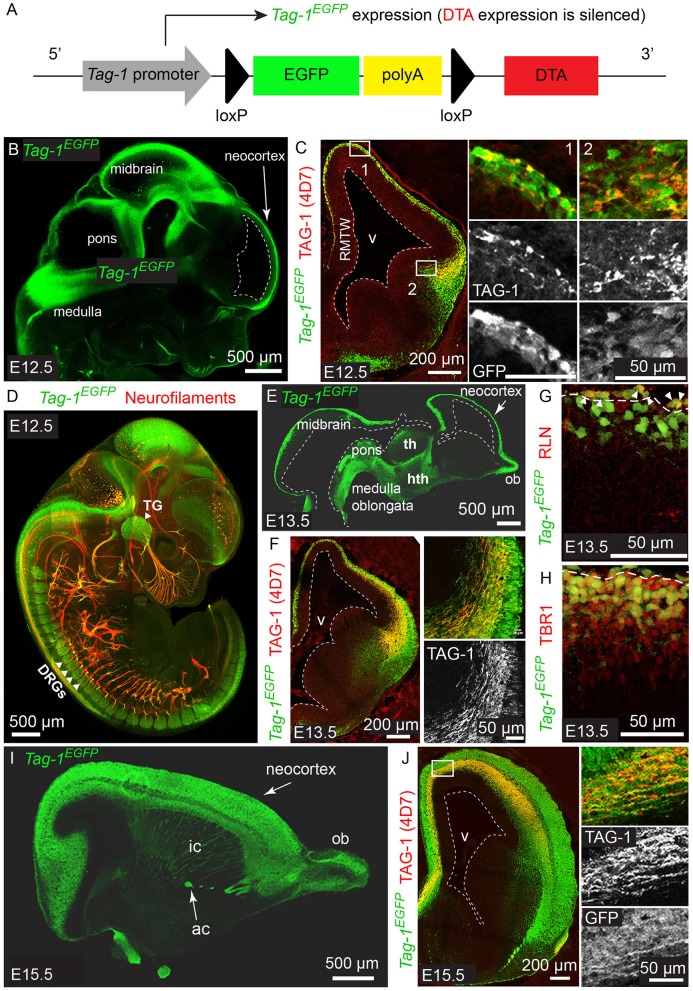
*Tag-1*^*EGFP*^ is expressed in the developing cortex, thalamus, internal capsule and brainstem and colocalizes with endogenous TAG-1. **(A)** Overview of *Tag-1*^*loxP*−*EGFP*−*loxP*−*DTA*^ transgene structure, which drives *Tag-1*^*EGFP*^ expression without interfering with endogenous *Tag-1* expression. **(B,C)** Immunofluorescent analysis of *Tag-1*^*EGFP*^ and TAG-1 (clone 4D7) expression in the mouse brain at E12.5. Note the coincidence of EGFP and TAG-1 signal in the early cortex and the axons of the cortical plate neurons. **(D)** Whole mount immunofluorescence against *Tag-1*^*EGFP*^ and neurofilaments (clone 2H3) on a representative E12.5 embryo. Note the axons derived from EGFP+ cells in the cranial ganglia and dorsal root ganglia. **(E,F)** Immunofluorescence against *Tag-1*^*EGFP*^ and TAG-1 (clone 4D7) on cryosections of the developing brain of representative E13.5 embryos. Note the coincidence of the EGFP and TAG-1 immunofluorescence on the developing corticothalamic axons extending to the striatum. **(G,H)** Immunofluorescence against EGFP and RLN or TBR1, respectively, in the cortex at E13.5. Note the presence of EGFP+/RLN+ cells (shown by arrowheads) at the marginal zone and EGFP+/TBR1+ cells at the marginal zone and cortical plate. **(I,J)** Immunofluorescent analysis of *Tag-1*^*EGFP*^ and TAG-1 (clone 4D7) expression in the mouse brain at E15.5. RMTW, rostromedial telencephalic wall; TG, trigeminal ganglion; DRGs, dorsal root ganglia; th, thalamus; hth, hypothalamus; ob, olfactory bulb; ic, internal capsule; ac, anterior commissure; v, ventricle.

Genetically modified *Tag1*^*loxP*−*GFP*−*loxP*−*DTA*^ mice were generated using BAC technology as described before (Bastakis et al., 2015). In brief, we used a BAC clone containing the *Tag-1* gene and a plasmid containing the appropriate homologous domains in order to induce recombination replacing the second exon of the *Tag-1* gene. The final BAC clone carried the *Tag-1* promoter, followed by a floxed eGFP-coding sequence followed by 4 SV40-polyA stretches that stop translation. Further downstream, we included the DTA-coding sequence (Figure 1A). As a result, these mice express GFP under the artificially introduced *Tag-1* promoter without affecting endogenous TAG-1 expression. In time-mated pregnancies the day of the vaginal plug detection was considered as E0.5 and the day the pups were born as P0. All mice used in this study were of the C57BL6/SV129 background. Housing and animal procedures used were according to the European Union policy (Directive 86/609/EEC) and institutionally approved protocols. PAC transgenic *Emx1-Cre* mice were obtained from Dr. F. Guillemot (Francis Crick Inst., London) (Fogarty et al., 2005; Kessaris et al., 2006). Upon crossing *Tag1*^*loxP*−*GFP*−*loxP*−*DTA*^ (from now on called *Tag-1*^*EGFP*^) with any Cre-expressing strain, Cre-mediated excision of the GFP-coding sequence results in GFP ablation and DTA expression. To obtain the experimental *Emx1*^*Cre*+^*; Tag1*^*loxP*−*GFP*−*loxP*−*DTA*+^ mice and to ensure we always worked with one copy of either *Cre* or DTA, we always crossed *Emx1-Cre*+ mice with *Tag1*^*loxP*−*GFP*−*loxP*−*DTA*^ mice. The hemizygous for both alleles *Emx1*^*Cre*+^*; Tag1*^*loxP*−*GFP*−*loxP*−*DTA*+^ mice will be called from now on *Emx1*^*Cre*^*; Tag1*^*DTA*^.

### Immunofluorescence

Embryos or dissected brains were collected and fixed in 4% paraformaldehyde in 1xPBS (pH 7.4) at 4°C for 24 h followed by three 1xPBS washes. In the case of processing for cryosections, samples were cryoprotected by incubating at 4°C overnight in 30% sucrose in 1xPBS. Tissue samples were subsequently embedded in gelatin/sucrose in 1xPBS, frozen at −20°C and sectioned at 10–16 μm thickness. Immunofluorescent detection on the obtained cryosections was performed as described previously (Kastriti et al., 2019).

### Primary Antibodies

The following antibodies were used:

Goat anti-GFP (1:500, #ab6662, Abcam), mouse anti-neurofilament (1:100, clone 2H3, DSHB), mouse anti-ISL1 (1:250, clone 39.4D5-s, DSHB), rat monoclonal anti-L1 (1:1,000, Clone 557.B6); (Appel et al., 1995), rat anti-L1 (1:1,000, MAB5272, Millipore), rat anti-GFP (1:1,000, #04404-84, Nacalai Tesque,), rabbit anti-GFP (1:5,000, Minotech), rabbit anti-neurofilament heavy (1:1,000, #ab8135, Abcam), chicken anti-neurofilament heavy (1:1,000; #ab4680, Abcam), rabbit polyclonal anti-TBR1 (1:600, #ab31940, Abcam), rat anti-BrdU (1:1,000,#OBT0030CX, AbD Serotec), mouse anti-TAG-1 (1:1,000, clone 4D7, DSHB) mouse anti-SATB2 (1:250, #ab51502/100, Abcam), rat anti-CTIP2 (1:1,000, # ab184650, Abcam), rabbit anti-cleaved caspase3 (1:200, # 9661, Cell Signaling), mouse anti-REELIN (1:200, clone G10, DSHB), chicken anti-GFP (1:500, GFP-1020 /Aves Labs Inc), goat anti-NURR1 (1:100, AF2156, R&D), rabbit anti-CPLX3 (1:1,000, Synaptic Systems), rabbit anti-BLBP (1:500 #ab32423, Abcam), rabbit anti-PVA (1:800, PV27, Swant), mouse anti-NEUN (1:100, MAB377, Millipore).

When TO-PRO3 was used, it was diluted to 1 μM and applied on sections for 3 min at room temperature, after immunohistochemistry was complete.

For detection of primary antibodies against goat, rabbit, mouse, and rat, secondary antibodies raised in goat or donkey and conjugated with Alexa-488,−555, and−647 fluorophores were used (1:800, Molecular Probes, ThermoFisher Scientific). For detection of primary antibodies raised in chicken, secondaries raised in donkey and conjugated with Alexa-488 or Cy3 fluophores were used (1:600, Jackson ImmunoResearch).

Whole mount fluorescent immunostaining of E12.5 mouse embryos, 3D imaging, visualization, and analysis were performed as previously described (Adameyko et al., 2009; Kastriti et al., 2019). After incubation with secondary antibodies and washing steps, embryos were cleared using BABB solution (1 part of benzyl alcohol/2 parts of benzyl benzoate) for a minimum of 1 h at room temperature with rotation prior to imaging.

### BrdU Birth-Dating of Newly Generated Neurons

BrdU injection and immunofluorescent detection were performed as previously described (Vidaki et al., 2012). Briefly, pregnant mice were injected on the appropriate day of gestation with 75 μg of BrdU diluted in PBS per g of body weight. Embryos were collected and processed for cryosections as explained above (see Immunofluorescence section).

### Quantification of Neuronal Populations and Bin Analysis

For the analysis of the EGFP+, SATB2+, CTIP2+, TBR1+, and BrdU+ cells and subplate cells, areas of the same cortical width of the somatosensory cortex were quantified in both genotypes. Cortical bins were defined by dividing the total height of the cortical column ranging from layer II to VIb (subplate—SP) in six equal segments (**Figure 3C**, cortical bin No 1 corresponding always to layer I or marginal zone and cortical bins 2–6 were bins of the same height, No2 the most superficial and no 6 to the closest to the ventricular zone). Even though we chose to split the cortical column in 6 bins, it is worth noting that these cortical bins 2–6 do not correspond to layers II–VIb but they are a rough approximation of the layered cortical structure.

### DiI Experiments

Tissue samples collected for DiI tracing (E16.5, E17.5, and P0 dissected whole brain) were fixed in 4% PFA in 1xPBS at 4°C, overnight. For anterior olfactory nuclei (AON) tracing experiments, following washes, a single DiI crystal was directly inserted in the tissue (P0 whole brain). For tracing of corticothalamic and other subcerebral axonal tracts, the whole brain was embedded in 2% low-melting agarose in 1xPBS and sectioned in a vibrating tissue slicer (Leica) in 100 μm-thick sections. Then, the DiI crystal was inserted in deeper cortical layers (layers V and VI) for corticofugal axonal tracing or in the lateral part of the thalamus for TCA tracing. Following DiI placement, tissue was kept in 1xPBS at 37°C for 7 days before imaging.

### Microscopy and Image Analysis

Imaging took place using TCS SP2, TCS SP8 (Leica Microsystems GmbH), and LSM710 (Zeiss) confocal microscopes equipped with a 10 ×, 20 ×, and 40 × objective. Raw lsm files were converted to either tiff or jpeg using the ImageJ software and final figures were designed using the Adobe Photoshop and Adobe Illustrator software.

### Quantification and Statistics

Data analysis and statistical tests were performed using GraphPad Prism 7, expressed as mean ± standard error of mean and statistical significance was calculated using 2-sided Student *t*-tests. Degree of significance was represented as follows: ns (non-significant)—*p* ≥ 0.05, ^*^p ≤ 0.05; ^**^p ≤ 0.01; ^***^p ≤ 0.001; ^****^p ≤ 0.0001.

### Adult Brain Preparation and Contrasting for Micro-Computed Tomography (μCT)

Adult mice were anesthetized and transcardially perfused with ice-cold 4% PFA to ensure efficient fixation. The brain was then dissected out of the skull and post-fixed for 1 h in 4% PFA with rotation at 4°C. Following three washes with cold 1xPBS, the tissue was gradually dehydrated through an ethanol gradient (30, 50, 80, 90%) and then transferred to 90% methanol (each step for 24–36 h at 4°C). Tissue contrasting was performed by incubation in 1.5% phospho-tungstic acid (PTA) in 90% methanol for 1 month at 4°C, while replacing the PTA solution once every week. For enhanced staining of the axonal tracts, a subsequent contrasting step was introduced with incubation in 2% iodine in 90% ethanol for 3 days at 4°C. Following contrasting, rehydration of the samples was performed by incubation in ethanol series (90, 70, 50, and 30%) and shipped to the CT-laboratory for scanning.

### Micro-Computed Tomography (μCT) Measurement and Data Processing

Adult brains dissected from the skull were embedded in 1% agarose gel in a polypropylene conical centrifuge tube for the purpose of motion stabilization during the micro CT scan.

The micro CT scan was performed using a laboratory system GE Phoenix v|tome|x L 240 (GE Sensing & InspectionTechnologies GmbH, Germany), equipped with a 180 kV/15 W maximum power nanofocus X-ray tube and high contrast flat panel detector flat panel dynamic 41|100 4,000 px ×4,000 px with 100 μm × 100 μm pixel size. The measurement was carried out in the air-conditioned cabinet (21°C). Each sample was scanned separately at acceleration voltage of 60 kV, X-ray tube current of 200 μA, exposure time 700 ms and 1,900 projections were taken over 360° resulting in 7 μm voxel resolution. The tomographic reconstruction of acquired data was realized by GE phoenix datos|x 2.0 software (GE Sensing & Inspection Technologies GmbH, Germany).

Data processing was realized according to Tesarová et al. (2016): The reconstructed tomographic data were imported in Avizo 9.5.0 software (ThermoFisher Scientific, USA) where manual segmentation of the corpus callosum (CC) and anterior commisure (AC) was carried out. The segmented data were then imported into VG Studio MAX 3.2 software (Volume Graphics GmbH, Germany) and the Sphere method of wall thickness analysis of the CC and AC was implemented.

## Results

### Characterization of Neuronal Populations Expressing TAG-1 in the Developing Cortex

According to previous studies, TAG-1 expression appears first in the PP of the embryonic cortex at E12.5 and by E14.5 it is expressed by the axons running through the intermediate zone, longitudinally across the cortex (Wolfer et al., 1994; Denaxa et al., 2001). Additionally, the protein has been shown to be released extracellularly due to its peripheral association with membranes (Furley et al., 1990; Karagogeos et al., 1991; Stoeckli et al., 1991; Savvaki et al., 2010), which renders its detection using immunocytochemistry technically challenging. We generated the mouse strain *Tag-1*^*loxP*−*EGFP*−*loxP*−*DTA*^ to further study the expression and role of TAG-1 in the developing embryo. In this transgenic mouse strain, EGFP is expressed under the promoter of *Tag-1*, while the transgene also carries in frame the sequence coding for DTA in quiescence (Diptheria Toxin subunit A; Figure 1A).

**Figure 2:**
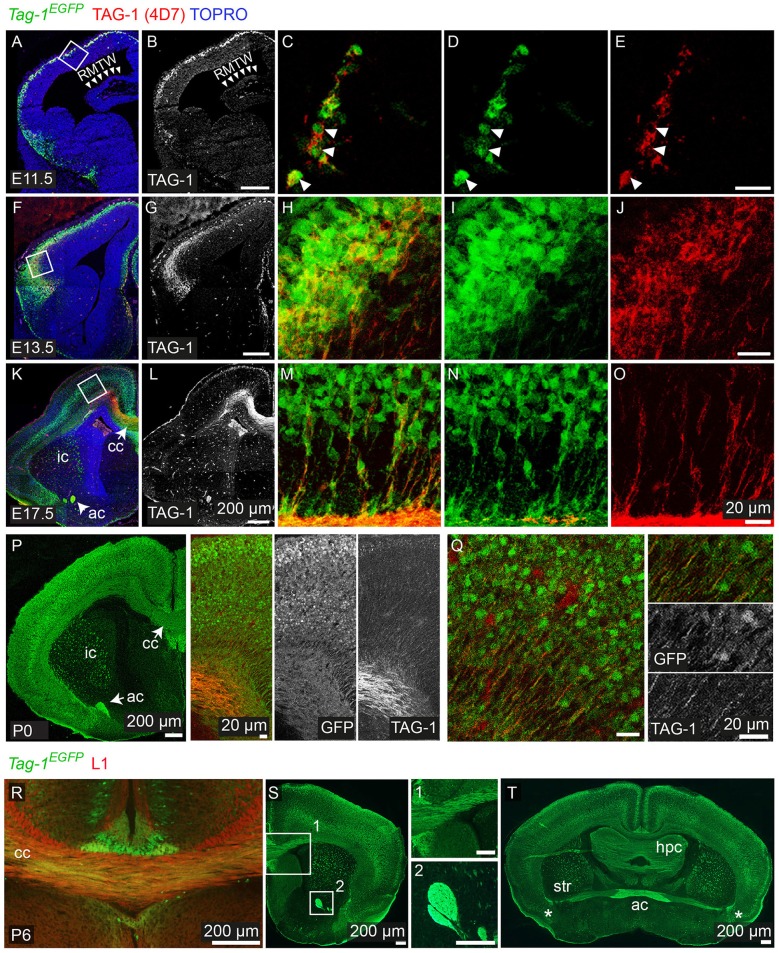
*Tag-1*^*EGFP*^ expression in the developing embryonic and early postnatal cortex. **(A–O)** Immunofluorescent analysis of *Tag-1*^*EGFP*^ and TAG-1 (clone 4D7) expression in the mouse brain during embryonic stages E11.5 to E17.5. **(P,Q)** Immunofluorescent analysis of *Tag-1*^*EGFP*^ and TAG-1 (clone 4D7) expression in the newly-born mouse brain. **(R–T)** Immunofluorescent analysis of *Tag-1*^*EGFP*^ and L1 in P6 mouse brain shows the composition of the corpus callosum and anterior commissure of *Tag-1*^*EGFP*+^ axons. Note the coincidence of EGFP and TAG-1 signal in the cortex and the associated axonal tracts. RMTW, rostromedial telencephalic wall, cc, corpus callosum, ic, internal capsule; ac, anterior commissure, str, striatum, hpc, hippocampus.

**Figure 3:**
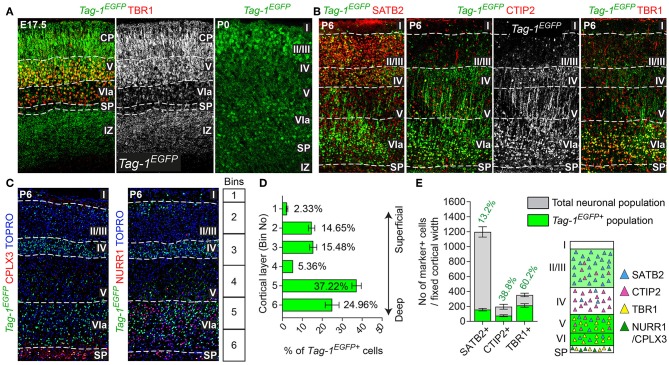
TAG-1+ neocortical neurons contribute to a small degree to upper layers and to a greater extent to deeper cortical layers postnatally. **(A)** Immunofluorescent analysis of *Tag-1*^*EGFP*^ and TBR1 in the cortex at E17.5 and P0. Note the reduced EGFP detection in the subplate (SP). **(B)** Immunofluorescence showing the expression of the *Tag-1*^*loxP*−*EGFP*−*loxP*−*DTA*^ transgene in the somatosensory cortex at P6 and the layer markers SATB2, CTIP2 and TBR1. **(C)** Immunofluorescent analysis of *Tag-1*^*EGFP*^ expression in the somatosensory cortex at P6 in combination with the subplate markers NURR1 and CPLX3. **(D)** *Tag-1*^*EGFP*+^ cell distribution across cortical layers. Data are plotted as mean ± SEM, *N* = 4. **(E)** *Tag-1*^*EGFP*+^ contribution to the SATB2+, CTIP2+, and TBR1+ neuronal populations of the P6 neocortex. Data are plotted as mean ± SEM, for SATB2: *N* = 4, for CTIP2: *N* = 3, for TBR1: *N* = 5 and the double positive EGFP+;marker+ population is shown superimposed on the total numbers of marker+ cells. The scheme is summarizing the distribution of *Tag-1*^*EGFP*+^ cells in the early postnatal neocortex. CP, cortical plate, SP, subplate, IZ, intermediate zone.

We analyzed the expression pattern of *Tag-1*^*EGFP*^ during embryogenesis, starting from E11.5. At that age, *Tag-1*^*EGFP*^ was detected in very few cells of the PP, similarly with endogenous TAG-1 (Figures 2A–E), which increased by E12.5 (Figures 1B,C). At E12.5, EGFP expression was also observed at the developing midbrain, medulla oblongata and pons in the CNS (Figures 1B,D and as shown previously Yamamoto et al., 1986; Wolfer et al., 1994). In the PNS, we were able to detect *Tag-1*^*EGFP*^ in the neurons of the trigeminal ganglion and dorsal root ganglia (DRG) and associated axons (Figure 1D), as reported for TAG-1 (Dodd et al., 1988; Wolfer et al., 1994). At E13.5, *Tag-1*^*EGFP*^ expression was similar to that at E12.5 in the cortex (cortical plate—CP) and further detected in the developing thalamus and hypothalamus, as well as the axons extending toward the striatum (Figures 1E,F, 2F–J). Additionally, at E13.5, we identified an EGFP+/RLN+ and an EGFP+/TBR1+ population, corresponding to MZ and cortical neurons, respectively, as described previously (Figures 1G,H; del Rio et al., 1995; Hevner et al., 2001; Espinosa et al., 2009). At E15.5, EGFP expression was detected in CP neurons, as well as in axons comprising the internal capsule (IC) (Figures 1I,J). Two days later, at E17.5, EGFP expression was observed in the CP, layers V and VI and axonal tracts such as the corpus callosum (CC) and IC (Figures 2K–O, 3A) but was detected in very few cells in the subplate (SP) (Espinosa et al., 2009; Figure 3A). It must also be noted that in all embryonic stages analyzed, very few EGFP+ cells were observed in the SP.

Overall, we observe that EGFP colocalizes with endogenous TAG-1 in the cortex during earlier developmental stages (E11.5 and E12.5). However, during later stages TAG-1 immunoreactivity is diminished compared to that for EGFP+ (Figures 1J, 2K–O), possibly due to the secreted nature of the protein (Furley et al., 1990; Karagogeos et al., 1991; Stoeckli et al., 1991; Savvaki et al., 2010). Upon birth, EGFP+ cells are found across the cortex and are progressively restricted to deeper cortical layers (Figures 2P,Q, 3A).

Thus, when taking in consideration the EGFP expression pattern we can conclude that the protein is expressed originally in the PP, then in the developing CP and lastly by neurons (and their axons) residing at all cortical layers during late embryogenesis, much more extensively than previously thought. Following birth and upon the initiation of maturation of pyramidal neurons it is mainly expressed by neurons residing in deeper layers of the cortex, which was undetectable until now with the currently available antibodies against TAG-1.

### *Tag-1^*EGFP*^* Cells Are Widespread in the Postnatal Cortex and Contribute to Both Upper and Deeper Layers

Given the observed dynamic expression of *Tag-1*^*EGFP*^ in the cortex during early postnatal stages, we first analyzed the contribution of *Tag-1*^*EGFP*^ cells along the layers of the somatosensory cortex at P6. At this time, the specification of all cortical layers is considered to be complete, as the late-born neurons of layer II/III are generated between E15.5 and E17.5 (reviewed by Molyneaux et al., 2007). We found that *Tag-1*^*EGFP*^ cells are distributed in all cortical layers, with the majority (92.31% of all EGFP+ cells) distributed between layers II/III and V/VI (30.13 and 62.18%, respectively of all EGFP+ cells; Figures 3B,D). However, only 2.33 and 5.36% of all EGFP+ cells were found in layers I and IV, respectively. The neurons of layers II/III correspond mainly to callosal neurons, while those of layers V and VI to corticofugal (subcerebral) neurons. The observation of specific enrichment of the transgene in layers II/III and V/VI urged us to analyse further the molecular profile of *Tag-1*^*EGFP*^ cells. We found that EGFP+ cells contribute by 60.2% to the TBR1+ population (corticofugal neurons, mainly populating layers V and VI; Bedogni et al., 2010; Han et al., 2011; McKenna et al., 2011) and by 38.8% to the CTIP2+ population (neurons projecting to subcerebral targets, mainly in layers IV and V, Arlotta et al., 2005; Chen et al., 2008; Figures 2B,E). Additionally, EGFP+ cells contributed by 13.2% to the total SATB2+ population of the cortex (found across the cortex, mainly enriched at layers II/III but also found in deeper layers and contributing to the specification of callosal and subcerebral neurons (Alcamo et al., 2008; Britanova et al., 2008; Srinivasan et al., 2012; Srivatsa et al., 2014; Leone et al., 2015; McKenna et al., 2015; Figures 2B,E). Lastly, analysis of the postnatal SP at P6 through the markers NURR1 and CPLX3 (Hoerder-Suabedissen et al., 2009; Hoerder-Suabedissen and Molnar, 2013) showed 17.6 and 8% contribution of *Tag-1*^*EGFP*+^ cells per marker, respectively (Figures 3C, **7C**).

Thus, taking these results together, *Tag-1*^*EGFP*^ expression in the cortex is maintained postnatally by pyramidal neurons that project not only to the contralateral hemisphere and thalamus, but also to other subcortical targets.

### *Emx1-Cre*–Induced Recombination in the Embryonic Cortex Results in *Tag-1^*DTA*^* Expression and Cell Death With Major Macroscopic Cortical Defects

**Figure 4:**
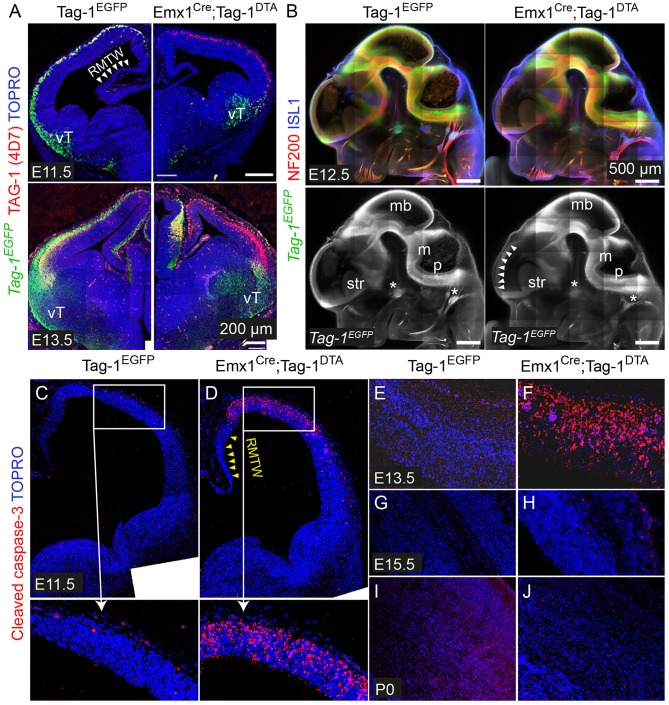
*Emx1*^*Cre*^-mediated recombination in *Tag-1*^*loxP*−*EGFP*−*loxP*−*DTA*^ embryos results in EGFP removal and neocortical cell death during stages E11.5 to E13.5. **(A)** Immunofluorescence against EGFP and TAG-1 in in *Tag-1*^*EGFP*^ and *Emx1*^*Cre*^*;Tag-1*^*DTA*^ mice at E11.5 and E13.5. Note the lack of recombination at the ventral telencephalon (vT). **(B)** Whole mount immunofluorescence against EGFP, neurofilaments and ISL1 in control and double transgenic mice at E12.5 shows the selective EGFP switch-off in the dorsal telencephalon (see white arrowheads). Note that sensory ganglia (ISL1+, shown by asterisks) remain unaffected upon Emx1^Cre^-mediated recombination. **(C–J)** Immunofluorescence against cleaved caspase-3 (reflecting cell death) in in *Tag-1*^*EGFP*^ and *Emx1*^*Cre*^*;Tag-1*^*DTA*^ mice at E11.5 to P0. RMTW, rostromedial telencephalic wall, vT, ventral telencephalon, mb, midbrain, m, medulla, p, pons, str, striatum.

**Figure 5:**
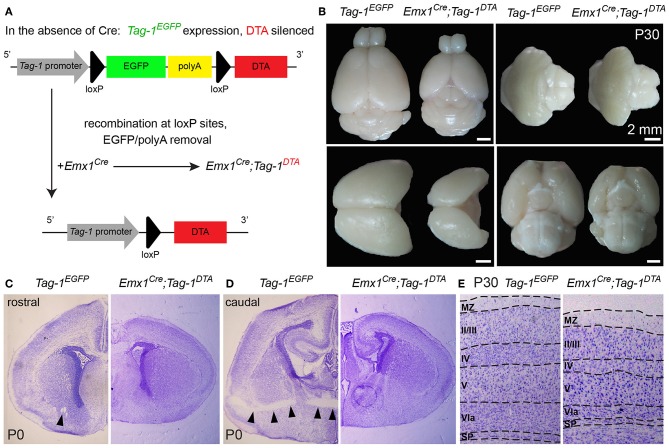
Macroscopic cortical defects in postnatal *Emx1*^*Cre*^*;Tag-1*^*DTA*^ mice. **(A)** Schematic showing the Cre-mediated recombination of the *Tag-1*^*loxP*−*EGFP*−*loxP*−*DTA*^ locus and the expression of DTA in the cortex, with simultaneous removal of EGFP but no effect on *Tag-1* expression. **(B)** Macroscopical examination of 1-month-old brain of *Tag-1*^*EGFP*^ and *Emx1*^*Cre*^*;Tag-1*^*DTA*^ mice, showing the decrease in size of the neocortex, while the cerebellum, pons and hypothalamus remain unaffected. **(C,D)** Cresyl violet morphological stain at a rostral **(C)** and caudal **(D)** level of the forebrain in *Tag-1*^*EGFP*^ and *Emx1*^*Cre*^*;Tag-1*^*DTA*^ mice. Note the absence of the anterior commissure in *Emx1*^*Cre*^*;Tag-1*^*DTA*^ mice, which is shown by black arrowheads in the control. No differences were observed in the thalamus or subpallium. **(E)** Cresyl violet morphological stain of 1-month-old brain show decrease in cortical thickness in *Emx1*^*Cre*^*;Tag-1*^*DTA*^. MZ, marginal zone; SP, subplate.

To investigate the role of TAG-1+ neurons of the developing cortex, we crossed the *Emx1-Cre* and *Tag-1*^*loxP*−*EGFP*−*loxP*−*DTA*^ strains (these mice will be referred to as *Emx1*^*Cre*^*;Tag-1*^*DTA*^ mice from now on, Figure 5A). Based on published *in situ* data (Lein et al., 2007), the two genes are co-expressed in the PP as early as E11.5 and E13.5, while *Emx1* expression is absent at later stages in the cortex (data not shown, refer to Allen Brain Atlas). In *Emx1-Cre* mice, Cre recombinase is mainly expressed by telencephalic neuronal progenitors and differentiated pyramidal neocortical neurons as early as E10.5 (Kessaris et al., 2006; Chou et al., 2009) and thus, upon crossing, results in the removal of EGFP and expression of DTA in TAG-1+ neurons leading to their death. Upon crossing, we observed the removal of EGFP in the cortex at E11.5 and E13.5 (Figure 4A). Examination of whole E12.5 embryos revealed EGFP removal specific to the cortex (Figure 4B). Macroscopically, we observed a severe decrease in the cortical thickness and overall size in *Emx1*^*Cre*^*;Tag-1*^*DTA*^ mice, but not in the cerebellum or pons at 1-month-old mice, domains where *Emx1* is not expressed (Figures 5A,B). Analysis of cleaved caspase-3 immunoreactivity revealed cell death specifically in the cortex from E11.5 to E13.5, while the striatum, cranial sensory ganglia, the developing midbrain, medulla and pons were unaffected (Figures 4C–F). Although extensive cell death was observed, we may assume that a minority of TAG-1+ cells escaped either because of lack of Cre expression or of low or no expression of *Tag-1* at the time of recombination. After E13.5, we did not observe any signs of cell death in the cortex during embryonic development nor early postnatal stage (P0) (Figures 4G–J), in agreement with the *in situ* data showing that *Emx1* is not expressed in the developing cortex after E13.5 (Lein et al., 2007). Moreover, newborn (P0) and 1-month-old mice showed reduced cortical thickness and abnormalities in the formation of the anterior commissure (AC) (Figures 5C–E), as described in detail below. Other abnormalities may include the morphology of the corpus callosum (CC) and hippocampus but were difficult to assess due to the significant malformation of the cortical region, as evident macroscopically (Figures 5B–D).

**Figure 6:**
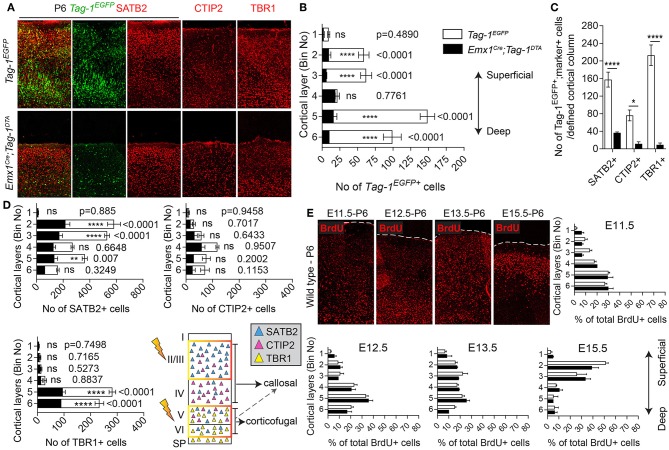
Targeted ablation of TAG-1+ cortical neurons results in neuronal reduction throughout the cortex. **(A)** Immunofluorescent analysis of the expression of the *Tag-1*^*loxP*−*EGFP*−*loxP*−*DTA*^ transgene in the somatosensory cortex at P6 and the layer markers SATB2, CTIP2, and TBR1 in *Tag-1*^*EGFP*^ and *Emx1*^*Cre*^*;Tag-1*^*DTA*^ mice. **(B,C)** Crossing of *Emx1*^*Cre*^ with *Tag-1*^*loxP*−*EGFP*−*loxP*−*DTA*^ and analysis at P6 results in a substantial decrease of *Tag-1*^*EGFP*+^ cells in layers II/III, V and IV, corresponding to all three SATB2+, CTIP2+, and TBR1+ subpopulations. In **(B)**, data are represented as mean ± SEM, *N* = 4 and 3 respectively, and the populations of EGFP+ cells in the *Tag-1*^*EGFP*^ and *Emx1*^*Cre*^*;Tag-1*^*DTA*^ somatosensory cortex are shown superimposed. In **(C)**, data are represented as mean ± SEM and respectively for *Tag-1*^*EGFP*^ and *Emx1*^*Cre*^*;Tag-1*^*DTA*^, for SATB2: *N* = 4 and 3, for CTIP2: *N* = 3 and 4, for TBR1: *N* = 5 and 4. **(D)** Upon ablation of TAG-1+ pyramidal neurons of the dorsal telencephalon, a significant decrease is observed in the total numbers of SATB2+ neurons of upper and deeper layers and CTIP2+ and TBR1+neurons of deeper layers. The scheme is depicting the layers which were the most affected upon ablation (orange frame). In **(D)**, data are represented as mean ± SEM with stacked values for *Tag-1*^*EGFP*^ and *Emx1*^*Cre*^*;Tag-1*^*DTA*^ and respectively for *Tag-1*^*EGFP*^ and *Emx1*^*Cre*^*;Tag-1*^*DTA*^, for SATB2: *N* = 4 and 3, for CTIP2: *N* = 3 and 4, for TBR1: *N* = 5 and 4. In **(C)**, for EGFP+;SATB2+ cells, *P* < 0.0001, for EGFP+;CTIP2+, *P* = 0.013 and for EGFP+;TBR1+, *P* < 0.0001. **(E)** BrdU detection and quantification in the somatosensory cortex of P6 mice following BrdU pulse-labeling at either E11.5, E12.5, E13.5, or E15.5 in *Tag-1*^*EGFP*^ and *Emx1*^*Cre*^*;Tag-1*^*DTA*^ mice. Data were plotted as mean ± SEM. Differences were not statistically significant (for pulses at E11.5, E12.5, and E15.5, *P* > 0.05, *N* = 3, for pulses at E13.5, *P* > 0.05, *N* = 2). ns, non-significant; *p < 0.05, **p < 0.01, ***p < 0.001, ****p < 0.0001.

Next, we analyzed the cellular organization in the brain of P6 *Emx1*^*Cre*^*;Tag-1*^*DTA*^ mice. We observed a dramatic decrease in total numbers of *Tag-1*^*EGFP*+^ cells in the somatosensory cortex of *Emx1*^*Cre*^*;Tag-1*^*DTA*^ mice in comparison to *Tag-1*^*EGFP*+^ (Figures 6A,B). The most severely affected cortical layers were layers II/III, V, and VI (Figure 6B), while the ablation of EGFP+ cells was found across all three EGFP+;SATB2+, EGFP+;TBR1+, and EGFP+;CTIP2+ populations (Figure 6C). Notably, EGFP+ cells of layer IV remained unaffected, as well as total numbers of single CTIP2+ cells (Figures 6B,D). Furthermore, when we quantified pyramidal neurons born at different time points from E11.5–E15.5, we detected similar percentages and distribution through layers in *Emx1*^*Cre*^*;Tag-1*^*DTA*^ cortex compared to controls (Figure 6E). This result suggests that the defect is not birthdate-specific and that the remaining cells in the *Emx1*^*Cre*^*;Tag-1*^*DTA*^ animals have properly migrated from the ventricular zone to their final destination.

**Figure 7:**
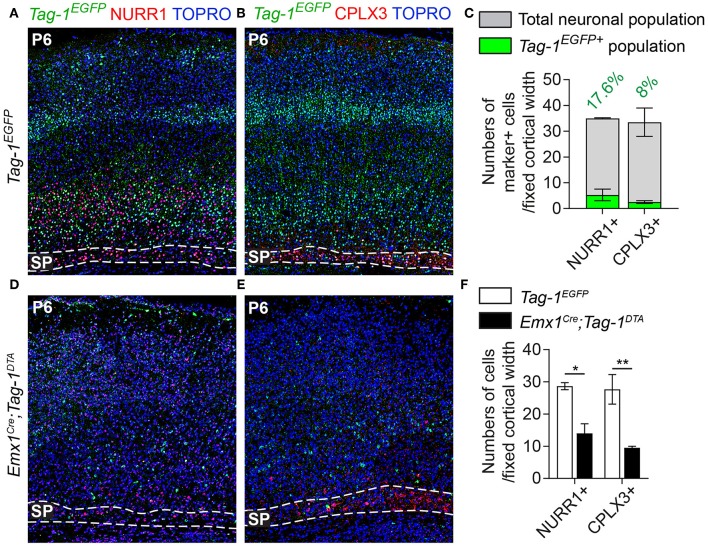
Targeted ablation of TAG-1+ cortical neurons results in reduced cells of the postnatal subplate. **(A,B,D,E)** Immunofluorescent analysis of *Tag-1*^*EGFP*^, NURR1, and CPLX3 at P6 in *Tag-1*^*EGFP*^ and *Emx1*^*Cre*^*;Tag-1*^*DTA*^ mice shows a detectable subplate at the somatosensory cortex. **(C)** Quantification of the contribution of *Tag-1*^*EGFP*+^ cells in the control subplate of the somatosensory cortex at P6. Data are plotted as mean ± SEM, *N* = 2. **(F)** Quantification of the cells of subplate in *Tag-1*^*EGFP*^ and *Emx1*^*Cre*^*;Tag-1*^*DTA*^ mice at P6 using the markers NURR1 and CPLX3. Data are plotted as mean ± SEM, *N* = 2. For NURR1+ cells, *P* = 0.019 and for CPLX3+ cells, *P* = 0.007. *p < 0.05, **p < 0.01.

Additionally, even though we found very few *Tag-1*^*EGFP*^ cells in the embryonic SP, the stage during which we observe cell death in the cortex, we examined this cortical layer at P6 and discovered that NURR1+ and CPLX3+ cells are affected significantly upon TAG-1+ neuronal ablation in the cortex (51.17% reduction of NURR1+ cells and 65.67% reduction of CPLX3+ cells; Figures 7D–F). Since we do not expect any cell death in the SP as an immediate result of the *Emx1-Cre*-mediated recombination during development, we hypothesize that the observed defects of the postnatal SP may be a secondary effect due to the loss of TAG-1+ axons.

### *Emx1-Cre*–Induced Decrease of TAG-1+ Neurons Resulted in Reduced Corticothalamic Axons and Callosal Defects

**Figure 8:**
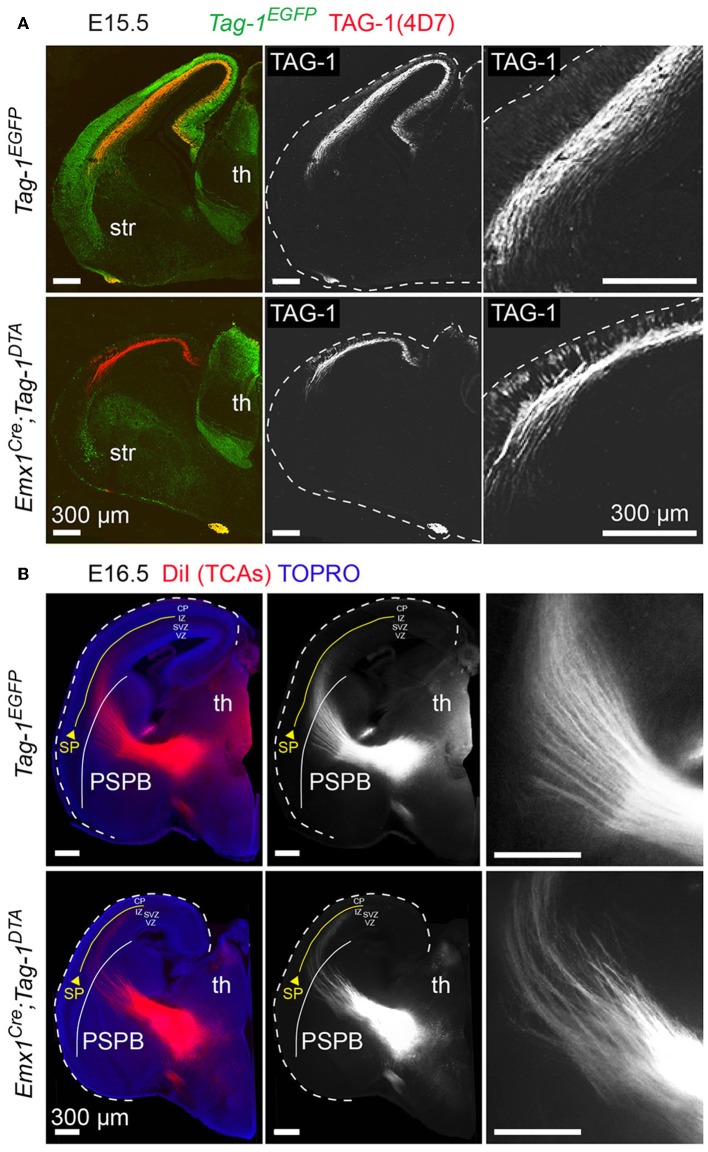
Targeted ablation of TAG-1+ neocortical neurons affects several axonal tracts in the forebrain but not the extension of thalamocortical axons. **(A)** Immunofluorescence against EGFP and TAG-1 in E15.5 forebrain in *Tag-1*^*EGFP*^ and *Emx1*^*Cre*^*;Tag-1*^*DTA*^ mice, showing the reduction in corticothalamic axons (CTAs) as shown by TAG-1. The edges of the cortex are marked by dashed lines. **(B)** Anterograde DiI tracing of thalamocortical axons (TCAs) on E16.5 brain in control and double transgenic mice showing normal development and extension of TCAs trough the pallium-subpallium boundary (PSPB, demarcated by the white line) even when the corticothalamic tract is severely reduced. Note that TCAs, as shown by the DiI tracing, are reaching the subplate (SP—demarcated by the yellow line). Th, thalamus, str, striatum, PSPB, pallial-subpallial boundary, SP, subplate, CP, cortical plate, IZ, intermediate zone, SVZ, subventricular zone, VZ, ventricular zone.

Since neocortical neurons of layers V and VI are reduced in *Emx1*^*Cre*^*;Tag-1*^*DTA*^ mice, corticothalamic axons (CTAs) should also be reduced. Indeed, a dramatic decrease, but not total absence, of TAG-1+ corticothalamic axons was observed at E13.5 and E15.5 (Figure 8A). It is known that corticothalamic and thalamocortical axons (TCAs) are a system of co-dependent axonal growth and fasciculation (Molnár et al., 1998a,b; Molnár and Blakemore, 1999; Hevner et al., 2002; Jones et al., 2002; Komuta et al., 2007; Chen et al., 2012; Deck et al., 2013). Thus, we reasoned that the decrease in CTAs could correlate with defective pathfinding of TCAs. To address that, we performed retrograde DiI tracing of the tract at E16.5, which showed that TCAs extend normally and reach the cortex in *Emx1*^*Cre*^*;Tag-1*^*DTA*^ mice (Figure 8B). Thus, in our model, partial ablation of CTAs of layers V and VI and decreased numbers of SP cells does not suffice to prevent TCA pathfinding and elongation.

**Figure 9:**
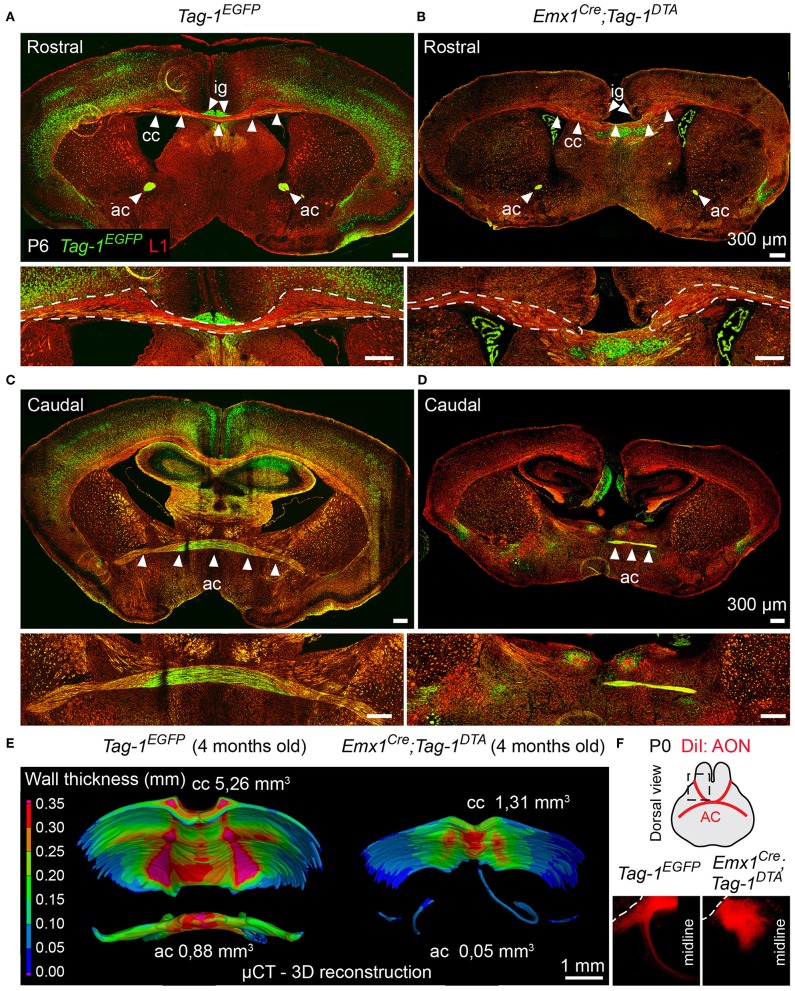
Targeted ablation of TAG-1+ neocortical neurons affects severely the corpus callosum and anterior commissure. **(A–D)** Immunofluorescence against EGFP and L1 on P6 forebrain in *Tag-1*^*EGFP*^ and *Emx1*^*Cre*^*;Tag-1*^*DTA*^ mice at a rostral **(A,B)** and caudal **(C,D)** level of the P6 forebrain in in *Tag-1*^*EGFP*^ and *Emx1*^*Cre*^*;Tag-1*^*DTA*^ mice reveals that the corpus callosum and anterior commissure (both structures depicted by white arrowheads) are greatly reduced upon ablation of TAG-1+ neocortical neurons. **(E)** Micro-computed tomography (μCT) of the adult brain (4 months old) and 3D reconstruction reveal a decrease in the thickness and overall reduction in the volume of the corpus callosum and a severely malformed anterior commissure in *Emx1*^*Cre*^*;Tag-1*^*DTA*^ mice. **(F)** Following anterograde DiI tracing on P0 dissected brain by DiI placement on the anterior olfactory nucleus (Luukko et al., 2008), it was visualized that the neurons of the AON are not projecting normally toward the midline in *Emx1*^*Cre*^*;Tag-1*^*DTA*^ mice, as compared to controls (*Tag-1*^*EGFP*^). Cc, corpus callosum, ac, anterior commissure, ig, indusium griseum, AON, anterior olfactory nucleus.

**Figure 10:**
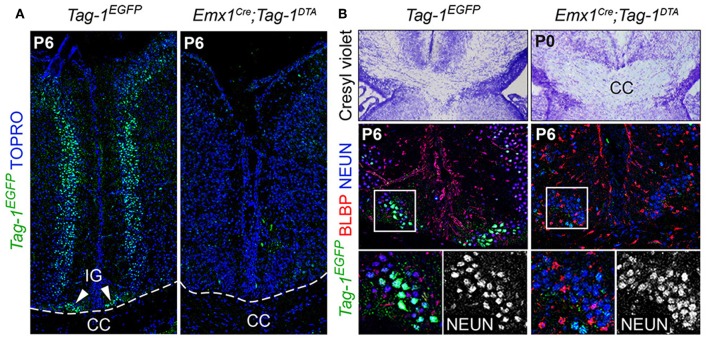
*Emx1-Cre* mediated ablation of TAG-1+ cells of the indisium griseum does not affect the guidepost cells of the corpus callosum. **(A)** Immunofluorescent analysis of *Tag-1*^*EGFP*^ at P6 in *Tag-1*^*EGFP*^ and *Emx1*^*Cre*^*;Tag-1*^*DTA*^ mice shows the ablation of a *Tag-1*^*EGFP*+^ cellular population at the midline (indusium griseum - IG). **(B)** Morphological staining and immunofluorescent analysis against *Tag-1*^*EGFP*^, BLBP and NEUN show no defect following the ablation of the *Tag-1*^*EGFP*+^ neuronal population at the midline in the vicinity of the corpus callosum. IG, induseum griseum, CC, corpus callosum.

Additionally, the decrease of EGFP+ cells in upper layers II/III, in combination with previous studies reporting the expression of TAG-1 by callosal axons (Yamamoto et al., 1986; Wolfer et al., 1994) and our observation of EGFP expression colocalizing with L1 staining in the postnatal corpus callosum (CC) (Figure 2R), as well as the decrease of EGFP+; SATB2+ cells, we proceeded to CC analysis. L1 immunostaining on P6 forebrain, revealed a defect in the CC axons (Figures 9A,B). Specifically, we observed an incomplete extension of the callosal axons toward the midline and an altered thickness on the mediolateral parts of the CC (Figures 9A,B). To exclude the possibility that the defect observed on the cryosections was just a regional abnormality, we did a follow-up experiment using micro-computerized tomography (μCT) on adult brains which revealed a dramatic difference in the overall volume of the CC accompanying the partial midline crossing of the midline (Figure 9E, Movies S1–S4). In search of the etiology of this defect, we assessed the potential structures that could be affected and, at the same time, take part in the development of the CC. Normal CC development depends on guidepost cells that guide the pioneer axons toward the midline, reviewed by Morcom et al. (2016), known as the indusium griseum (IG), which is composed by neurons and glia and a lateral mixed neuronal/glial population, known as the glial sling (Silver et al., 1982; Shu and Richards, 2001; Shu et al., 2003). Interestingly, the neurons of the IG are found in part in the *Emx1*+ domain (Niquille et al., 2009). Additionally, examination at P6 in *Tag-1*^*EGFP*^ mice revealed a GFP+ population coinciding with the IG, in which EGFP removal is observed following *Emx1-Cre*-mediated recombination (Figure 10A). As the IG is composed of glutamatergic neurons, interneurons and glial cells, we performed analysis using NEUN (to detect the total neuronal population) and BLBP (which marks astroglia) and observed no defect regarding any of the two populations (Figure 10B).

Thus, we conclude that the observed callosal defects are either due to the reduced number of SATB2+ neurons or to an extensive defect in pioneer callosal axons.

### *Emx1^*Cre*^;Tag-1^*DTA*^* Mice Show Postnatal Defects in the Anterior Commissure Formation

Another major axonal tract in the forebrain is the AC, which is composed of a rostral and a caudal component and is formed by the axons of neurons of the AON (Luukko et al., 2008), the olfactory tubercle, perirhinal cortex, agranular insular area and temporal cortex, and amygdaloid nuclei and the piriform cortex of the temporal lobes (Livy et al., 1997). Previous knowledge of TAG-1 expression by the AC, and specifically neurons of the AON (Yamamoto et al., 1986; Wolfer et al., 1994; Bastakis et al., 2015), ushered as to examine *Tag-1*^*EGFP*^ expression by the AC. Indeed, we detected EGFP+ axons in both components of the AC during late embryonic and early postnatal development (Figures 2K,P,S,T). Additionally, we observed a decrease in the thickness of the AC in early postnatal *Emx1*^*Cre*^*;Tag-1*^*DTA*^ mice (Figures 9C,D). Furthermore, DiI tracing from the AON clearly depicted the anterior AC in the brain of newborn control mice extending toward the midline, but failed to do so in the TAG-1-ablated brain (Figure 9F), confirming our previous observations. Analysis of adult brain using μCT showed an even more dramatic phenotype of misguidance of the AC (Figure 9E).

Thus, we conclude that *Tag-1*^*EGFP*^ mimics the reported expression for TAG-1 in the AC and that *Emx1*^*Cre*^-induced ablation of TAG-1+ neurons results in a significant reduction of the TAG-1+ neuronal population that contributes to the AC, with a small part of the tract still detectable.

## Discussion

TAG-1 (CNTN2) has been previously studied in some detail in the developing nervous system. Past studies have mapped TAG-1 expression in the developing forebrain, showing its expression in the PP, which later gives rise to the MZ and maintains TAG-1 expression (Denaxa et al., 2001). During subsequent development, TAG-1 is expressed by cortical neurons that extend their axons toward the thalamus (or corticothalamic axons—CTAs) through the intermediate zone and the axons of the internal capsule (Denaxa et al., 2001).

Among the members of the Contactin family, TAG-1 (CNTN2) is the sole protein expressed by the developing neurons and their axons in the forebrain during embryonic development, reviewed by Gennarini et al. (2017) and Oguro-Ando et al. (2017). Taking advantage of this unique expression, we created a mouse model expressing *Tag-1*^*EGFP*^ which would allow us to trace the expression of TAG-1 for a longer period and more effectively than the detection of the protein. Additionally, we wanted to assess the role of TAG-1+ fibers extending from the neocortex in the development of the cortex without affecting genetically the expression of any other axonal guidance molecule, which we approached experimentally by crossing this strain to the *Emx1-Cre* strain. This model comes with a few shortcomings, such as the partial ablation of the TAG-1+ population and the uncontrollable and rather late ablation of these neurons (as opposed to early ablation of the TAG-1+ cells already at E10.5, when the protein is firstly detected in the PP). However, it is a particularly useful model to study the effect of the loss of particular subpopulations of pyramidal neurons on the development of later-born neurons, as well as how would other axonal systems adapt to such major perturbations in the cortex, along with the CTAs, CC, and AC.

Additionally, several studies have shown that TAG-1 is also found in a secreted form, partially due to the fact that the protein is a GPI-anchored glycoprotein, pointing to multiple unknown functions that would be mediated through a paracrine mechanism in addition to the already described juxtacrine functions (Furley et al., 1990; Karagogeos et al., 1991; Stoeckli et al., 1991; Savvaki et al., 2010).

Previous studies in *Tag-1*^−/−^ mice did not reveal any obvious phenotype in the cortex and corticofugal axons (Denaxa et al., 2005), while the direct involvement of TAG-1+ neurons in shaping the developing telencephalon and establishing the identities of the various neuronal subpopulations has not been addressed thus far.

In our model, using the *Tag1*^*loxP*−*EGFP*−*loxP*−*DTA*^ transgene, we were able to analyse in detail the expression of *Tag-1* during embryonic and early postnatal development of the nervous system. Apart from showing the overlap between the expression of the transgene and the already published expression pattern of TAG-1 (as seen so far through immunohistochemistry), we observed for the first time a widespread and dynamic expression in cortical layers. Specifically, during embryonic development, *Tag-1*^*EGFP*^ was expressed by cortical plate cells and axons that were extending from the developing pyramidal neurons. However, following birth, *Tag-1* expression was gradually restricted mostly to deeper cortical layers and to a lesser degree in upper layers. This dynamic expression might correlate to the mechanisms of neuronal maturation and synaptic contact establishment.

In the peripheral nervous system during embryonic development, the protein is expressed by dorsal root ganglia (DRG) neurons and their axons, taking part in their elongation, pathfinding, and fasciculation (Yamamoto et al., 1986; Furley et al., 1990; Stoeckli et al., 1991; Wolfer et al., 1994; Masuda et al., 2000, 2003, 2004; Perrin et al., 2001; Law et al., 2008). In accordance with these studies, we also observed the expression of *Tag-1*^*EGFP*^ in the DRGs at E12.5, constituting the new transgene valuable for studies of the PNS.

When analyzing the postnatal somatosensory cortex, we found that *Tag-1* was expressed by SATB2+ and TBR1+ populations and *Tag-1*-mediated cell death of pyramidal neurons (peaking at E11.3–E13.5) resulted in a significant reduction in these total populations (39.6% for SATB2+ and 37% for TBR1+ cells; Figure 6C). Previous reports of transient TAG-1 expression by corticofugal and callosal axons during development (Yamamoto et al., 1986; Wolfer et al., 1994) are in accord with the expression of *Tag-1*^*EGFP*^ by TBR1+ and SATB2+ cells, respectively, between E14.5 and E16.5. However, especially in the case of postnatal and adult stages, the persistence of *Tag-1*^*EGFP*^ expression can be of great importance to further evaluate the role of TAG-1 in axonal development, maintenance and maturation.

Interestingly, even though the contribution of *Tag-1*^*EGFP*+^ cells in the CTIP2+ population (giving rise to subcortical projections) was significant (38.8%, Figure 3E), *Emx1-Cre*-mediated recombination leading to the expression of *Tag1*^*DTA*^ did not result in any significant effect in the total CTIP2+ population (Figure 6C). We hypothesize that this might be due to low expression levels of *Emx1-Cre* or *Tag-1* in these cells at the time of recombination. Interestingly, a recent study showed that CTIP2 is a powerful marker of interneurons in the adult somatosensory cortex (Nikouei et al., 2016). Taking this finding in consideration, it is also possible that *Tag-1*^*EGFP*+^;CTIP2+ cells correspond to interneurons, in which *Emx1-Cre* is not expressed. These results point to a potential expression of TAG-1 by interneurons and could prove of great interest for further studies (Bonetto et al., 2019). Alternatively, it is possible that a mechanism controlling the number of CTIP2+ cells could be participating to compensate the widespread neuronal loss of the SATB2+ and TBR1+ populations, a possibility that is worth exploring in future studies. An additional future perspective would be to also test experimentally the functionality of the neuronal circuits, in the case that such a mechanism would be proven to be in place.

An important observation was the significant reduction of NURR1+ and CPLX3+ cells of the early postnatal SP (Figure 7). This was a surprising finding, since *Tag-1*^*EGFP*^ was expressed by very few cells of the SP during embryonic stages, at time during which Emx1-Cre-mediated cell death took place. Furthermore, there is evidence showing that some SP cells are derived from a region outside the cortex, termed the rostromedial telencephalic wall (RMTW), a domain that lies outside the EMX1+ cortical area (Pedraza et al., 2014). Examination of the RMTW at E11.5 did not show any *Tag-1*^*EGFP*^ expression (Figures 2A,B) and, in addition, we did not observe any cell death in the RMTW in our model of ablation of the EMX1+;TAG-1+ cells (Figures 4A,C,D). Thus, we can conclude that the observed SP reduction is an indirect effect of the absence of TAG-1+ axons, which might be necessary for the migration of SP neurons in order to populate the developing cortex.

Furthermore, even though we did not observe *Tag-1*^*EGFP*^ expression in the embryonic SP, we detected a notable contribution of *Tag-1*^*EGFP*+^ cells to NURR1+ and CPLX3+ cells, a finding that could be important for studies targeting the postnatal SP.

In the adult rodent nervous system, there is knowledge of TAG-1 expression in the cerebellum, the mitral cells of the olfactory bulb, the hippocampus, and the juxtaparanodal domains of myelinated fibers (both at the axonal and glial membranes; Yoshihara et al., 1995; Wolfer et al., 1998; Traka et al., 2002). Considering the wider extent of detection of *Tag-1*^*EGFP*^ during the stages analyzed compared to the immunodetection with current antibodies, the transgenic strain in question is a valuable tool to further study the protein in the adult mouse (Bastakis et al., 2015; Zoupi et al., 2018). This could allow a variety of studies focusing on neurons, glia and potentially interneurons, as already seen in recent publications.

As mentioned above, *Emx1*^*Cre*^*;Tag-1*^*DTA*^-mediated cell death resulted in a reduction in upper layer SATB2+ neurons, which correspond to those giving rise to callosal axons, and to a reduced population of TBR1+ neurons of deeper layers, giving rise to corticothalamic axons. Following the targeted ablation of EMX1+/TAG-1+ neurons, CTAs showed an obvious reduction and callosal and anterior commissure axons were greatly affected.

Following previous knowledge of the co-dependence of CTAs and TCAs (Molnár et al., 1998a,b; Molnár and Blakemore, 1999; Hevner et al., 2002; Jones et al., 2002; Komuta et al., 2007; Chen et al., 2012; Deck et al., 2013), we tested the thalamocortical tract and found that it formed successfully and crossed the PSPB. Thus, we come to the conclusion that partial ablation of layer 5 and 6, but not embryonic SP CTAs does not suffice to prevent TCA pathfinding and elongation, although subtle defects in the TCA tracts could not be excluded. Finally, many studies have shown that the development and placement of SP cells is decisive for proper TCA extension and cortical integration, since TCAs always target the SP prior to postnatal CP invasion (Allendoerfer and Shatz, 1994; Herrmann et al., 1994; Molnár and Blakemore, 1995; Molnár et al., 1998b; Hoerder-Suabedissen and Molnar, 2015). Interestingly, even though in our model we have reduced numbers of CPLX3+ and NURR1+ cells in the postnatal SP, but with correct placement below the layer VIa, and we are not affecting the embryonic SP, it is not surprising that normal TCA invasion into this layer was observed (Figure 8B).

The defects in the development of the CC could be either a primary effect of the reduced SATB2+ neurons which give rise to callosal axons, or a secondary effect of the perturbation of guidepost cells involved in callosal development. Our analysis of the guidepost cells in the vicinity of the CC did not reveal any defect that could point to a secondary effect on callosal development, such as affected development of the IG or glial sling (Figure 10B). However, this was not surprising, since birth-dating experiments have shown that the guidepost cells of the CC are born after E14.5 (Shu et al., 2003), a developmental time during which we do not observe any more cell death following *Emx1-Cre*-mediated recombination (Figures 4G,H). One can also hypothesize that callosal axons extending from both hemispheres could constitute another case of co-dependent axonal extension that would require contact in the midline in order to further extend to the contralateral hemisphere.

Additionally, in *Emx1*^*Cre*^*;Tag-1*^*DTA*^ mice, we observed a dramatic reduction in both parts of the AC (both the anterior and posterior parts). In *Emx1*^*Cre*^*;Tag-1*^*DTA*^ mice, the tract was not completely absent, but significantly reduced, which might be explained by only partial expression of *Emx1* by the neurons giving rise to this tract. However, we observed further enhancement of the mutant phenotype with age, which may be due to the dependence of the axons of the AC on fasciculation for survival. Previous studies have shown that decrease in any axonal system of the brain is accompanied by defects (either reduction/atrophy or increased axonal numbers) in other axonal tracts (Livy and Wahlsten, 1997; Livy et al., 1997). The exact mechanisms underlying these phenomena are not known and their elucidation would be of great interest. By crossing with the appropriate Cre or CreERT strain with *Tag-1*^*loxP*−*EGFP*−*loxP*−*DTA*^, the ablation of a specific tract in the forebrain can be induced in order to study its effect on the other axonal systems.

Taken together, our results show that TAG-1+ neocortical cells contribute to the normal development of the cortex and the axonal tracts that form the corticothalamic tract, CC and AC. When TAG1+;EMX1+ neurons are removed, CTAs, the CC, and AC are significantly reduced with the mice exhibiting a plethora of other forebrain defects such as a potentially smaller hippocampus, potential altered striatal projections and some alterations in ventral telencephalon neurons. It would be of great interest for future studies to elaborate further (by means of electrophysiology and behavioral tests) on the effect that these major axonal defects have on the overall activity and functionality of the associated neuronal circuits, as well as the targets of the innervation of the cortex. The reduction, but not complete absence of CTAs was not sufficient to cause defects in the navigation of the TCA tract, which points to the fact that the presence of even a few of the axons navigating toward the thalamus are enough to guide efficiently the TCA axons through the pallial-subpallial boundary. Additionally, the described model resulting in targeted deletion of pyramidal neurons can constitute a useful tool to study the role of various neocortical subpopulations during development, as well as studies focused on the thalamus. Last but not least, the *Tag1*^*loxP*−*EGFP*−*loxP*−*DTA*^ transgenic strain can be of great value in the study of the peripheral nervous system, populations of glia, and potentially interneurons.

## Data Availability Statement

The raw data sets generated for this study are available upon request to the corresponding author.

## Ethics Statement

All work with mice took place under the approved protocol by our institutional review board (IRB) which is an independent Research Ethics Committee (REC) and by our Institutional Animal Care and Use Committee (IACUC) which has been approved by the Veterinarian Authorities Office (project license no. 93164) and was performed by trained scientists and by adherence to the 3R principles.

## Author Contributions

MEK, AS, DM, MT, MS, and KT designed, performed, and analyzed experiments. MK, TZ, and JK were involved in the μCT scans and reconstructions. IA provided reagents and advice for the whole mount immunofluorescence. MV generated the mouse line. DK supervised the work. MEK and DK wrote the paper. All authors provided valuable input on the manuscript content.

### Conflict of Interest

The authors declare that the research was conducted in the absence of any commercial or financial relationships that could be construed as a potential conflict of interest.

## Supplementary Material

Movie S1 Video of volume rendering 3D reconstruction of μCT data obtained from a 4-month-old *Tag-1*^*EGFP*^ mouse.

Movie S2 Video of volume rendering 3D reconstruction of μCT data obtained from a 4-month-old *Emx1*^*Cre*^*;Tag-1*^*DTA*^ mouse.

Movie S3 Video of coronal view of the axonal structures used for the volume rendering and 3D reconstruction of μCT data obtained from a 4-month-old *Tag-1*^*EGFP*^ mouse.

Movie S4 Video of coronal view of the axonal structures used for the volume rendering and 3D reconstruction of μCT data obtained from a 4-month-old *Emx1*^*Cre*^*;Tag-1*^*DTA*^ mouse.
